# Functional cyclophilin D moderates platelet adhesion, but enhances the lytic resistance of fibrin

**DOI:** 10.1038/s41598-018-23725-4

**Published:** 2018-03-29

**Authors:** Imre Varjú, Veronika Judit Farkas, László Kőhidai, László Szabó, Ádám Zoltán Farkas, Lívia Polgár, Christos Chinopoulos, Krasimir Kolev

**Affiliations:** 1 Department of Medical Biochemistry, Semmelweis University, Budapest, 1094 Hungary; 2 Program in Cellular and Molecular Medicine, Boston Children’s Hospital, Harvard Medical School, Boston, 02115 USA; 3 Department of Genetics, Cell- and Immunobiology, Semmelweis University, Budapest, 1089 Hungary; 4 Department of Functional and Structural Materials, Institute of Materials and Environmental Chemistry, Research Centre for Natural Sciences, Hungarian Academy of Sciences, Budapest, 1117 Hungary; 5 MTA-SE Lendület Neurobiochemistry Research Group, Budapest, 1094 Hungary; 6 Department of Sociomedical Sciences, Mailman School of Public Health, Columbia University, New York, NY 10032 USA

## Abstract

In the course of thrombosis, platelets are exposed to a variety of activating stimuli classified as ‘strong’ (e.g. thrombin and collagen) or ‘mild’ (e.g. ADP). In response, activated platelets adhere to injured vasculature, aggregate, and stabilise the three-dimensional fibrin scaffold of the expanding thrombus. Since ‘strong’ stimuli also induce opening of the mitochondrial permeability transition pore (MPTP) in platelets, the MPTP-enhancer Cyclophilin D (CypD) has been suggested as a critical pharmacological target to influence thrombosis. However, it is poorly understood what role CypD plays in the platelet response to ‘mild’ stimuli which act independently of MPTP. Furthermore, it is unknown how CypD influences platelet-driven clot stabilisation against enzymatic breakdown (fibrinolysis). Here we show that treatment of human platelets with Cyclosporine A (a cyclophilin-inhibitor) boosts ADP-induced adhesion and aggregation, while genetic ablation of CypD in murine platelets enhances adhesion but not aggregation. We also report that platelets lacking CypD preserve their integrity in a fibrin environment, and lose their ability to render clots resistant against fibrinolysis. Our results indicate that CypD has opposing haemostatic roles depending on the stimulus and stage of platelet activation, warranting a careful design of any antithrombotic strategy targeting CypD.

## Introduction

At the site of vascular injury, adhesive ligands (collagen, von Willebrand factor) are exposed that not only sequester platelets, but also contribute to their activation through specific receptors. The consequent exposure of phosphatidyl-serine promotes assembly of coagulation-activating complexes on the surface of platelets. This leads to generation of thrombin^1^, another potent platelet activator. Since the concentration of thrombin declines from the core to the periphery of the platelet plug^2^, peripheral plug expansion is dependent on the release of soluble ligands (e.g. ADP) from activated platelets^3^. The diversity and spatial gradients of activating signals result in distinct functional subpopulations of platelets within a single clot^1^. On one hand, platelets in the core region are exposed to activators such as thrombin, collagen or blood cell-derived reactive oxygen species (ROS). The combination of thrombin plus collagen (or its peptide analogue convulxin)^4^ as well as thrombin plus ROS are considered *‘strong’* stimuli that induce the formation of the mitochondrial permeability transition pore (MPTP)^5^. The platelet population exhibiting MPTP assembly is of primary importance regarding thrombin generation and subsequent fibrin formation with an impaired ability of aggregation^1^. These platelets lacking a unified characterisation have been described as ‘Sustained Calcium-Induced’^6^, ‘coated’^7^, ‘highly activated’^5^, ‘apoptotic’^8^, ‘procoagulant’^9^, and ‘necrotic’^10^. On the other hand,*’mild’* stimuli such as ADP induce platelet activation without MPTP formation, and are primarily relevant to the peripheral regions of clots. This is evidenced by the observation that inhibition of ADP-receptor signalling impairs platelet aggregation in the periphery of thrombi without affecting the core region^3^.

As MPTP is not a unique feature of platelet mitochondria, its modulation is of therapeutic interest in various aspects^11^. Its formation in nucleated cells may promote necrosis or apoptosis^12^, and is associated with pathological conditions such as reperfusion injury of the myocardium^13^ and brain^14^. Therefore, several attempts have been made to reveal its molecular identity. Recently, the interface within ATP synthase dimers^15^ and the c-rings of the ATP synthase^16^ have been favoured as structural components. Among its modulators, cyclophilin D (CypD), a peptidyl-prolyl cis-trans isomerase, has been long recognised as a major enhancer of MPTP opening^17^. Consequently, various investigations have been carried out to define the role of CypD in platelet activation^4,5,10,18–21^. While these studies have provided invaluable insight on the critical function of CypD in phosphatidyl-serine exposure and membrane receptor inactivation, a number of important details are still unclear. (i) Since these studies have been focusing on the role of CypD in the course of platelet MPTP formation, ‘strong’ activators were used. Therefore, the role of CypD in the course of ‘mild’ stimulus-induced platelet activation was not addressed. (ii) Many previous studies have utilised cyclosporine A (CsA) to bind CypD and delay MPTP formation^22^. However, CypD is not the only target of CsA. In fact, its Cyclophilin A (CypA)-mediated inhibitory effect on phosphoprotein phosphatase 2B (calcineurin) accounts for the use of CsA as an immunosuppressant^23,24^. A study reporting that calcineurin restricts platelet activation in a murine model highlights the relevance of the latter pathway^25^. Given the thrombotic complications of patients treated with CsA^26^, it is crucial to determine the overlaps and discrepancies of molecular pathways through which CsA and CypD affect platelet function. (iii) The effects of genetic ablation of CypD in *in vivo* thrombosis models are contradictory^5,10,20^. (iv) While the above cited *ex vivo* and *in vivo* studies provided insights on the role of CypD in the formation of thrombi, the effect of CypD on the platelet-driven modification of enzymatic clot digestion, *fibrinolysis* (reviewed by Longstaff & Kolev^27^) remains unexplored.

The present *ex vivo* study addresses these points and expands current knowledge in the field by revealing novel aspects of the role of CypD as well as the effects of CsA in platelet activation. In addition to ‘strong’ activators frequently used in previous studies, we have expanded our investigations using ADP, a stimulus that is considered ‘mild’, not known to cause MPTP formation. Besides aggregation assays using murine, CypD knockout (CypD^−/−^) and human, CsA-treated platelets, we introduce an impedance-based method to determine CypD and CsA effects on real-time platelet adhesion, and we address the effect of CypD on the lytic susceptibility of platelet-fibrin composite clots. To monitor changes in platelet structure, we apply a multi-faceted approach using scanning (SEM) and transmission electron microscopic (TEM) imaging of collagen-adherent, solution-suspended, and fibrin-entrapped platelets.

## Results

### CypD and CsA effects on the morphological alterations of platelets induced by ‘strong’ stimuli

**Figure 1:**
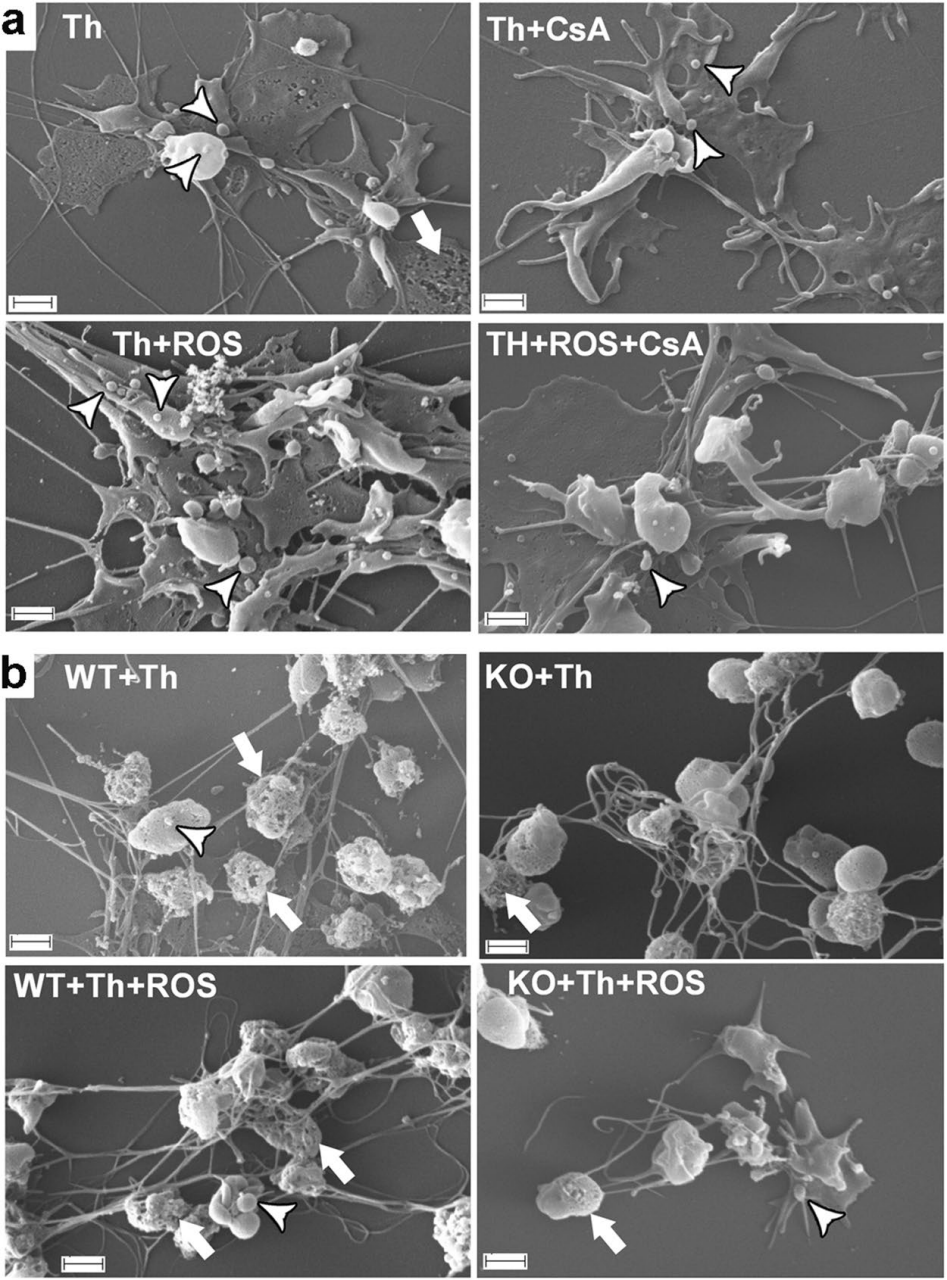
Adhesion and activation of platelets on collagen. (**a**) Washed human platelets and (**b**) wild type (WT) or CypD^−/−^ (KO) washed murine platelets were placed on collagen-coated glass surfaces and simultaneously activated with 25 nM thrombin in the presence of additives as indicated (ROS, CsA). SEM images were taken following fixation with glutaraldehyde. The arrowheads mark representative cases of membrane blebbing and shedding, the arrows point to membrane fragmentation and disintegration. Scale bar = 1 µm.

To mimic the environment at the site of vascular injury, platelets were allowed to adhere to a collagen surface, and soluble activators relevant to the core area of the platelet plug (thrombin and ROS) were added. ROS (hydroxyl radicals) were generated using physiological concentrations of Cu^2+^, homocysteine and H_2_O_2_, as described previously^28,29^. Human platelets activated by thrombin and collagen showed flattening and surface spreading, which were accompanied by membrane blebbing and fragmentation (e.g. Fig. 1a, upper left panel, lower right corner). In the presence of CsA, membrane integrity remained more preserved (Fig. 1a, upper right panel). In the presence of ROS, platelets exhibited dramatic shape changes and extensive shedding of membrane particles, and these effects were markedly counteracted by CsA treatment (compare lower left and right panels of Fig. 1a). Thrombin plus collagen-activated CypD^+/+^ murine platelets also exhibited spreading and membrane disintegration (Fig. 1b, upper left panel, lower region and middle region, respectively), while platelets isolated from CypD^−/−^ mice showed more preserved membrane integrity. Similarly, differences in membrane integrity, although less pronounced, could be noted between platelets of different genotype in the presence of ROS (compare lower left and right panels of Fig. 1b).

**Figure 2:**
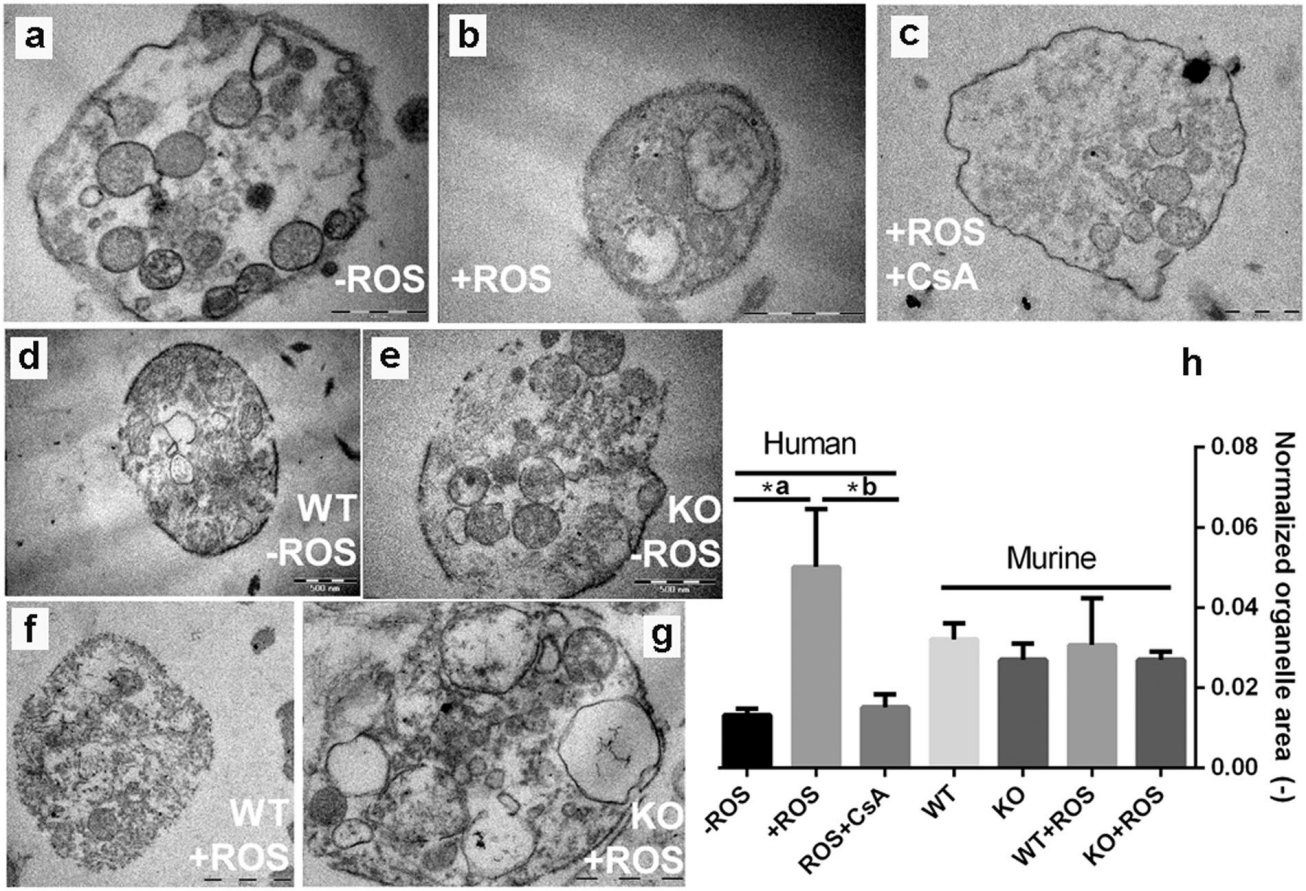
Effects of reactive oxygen species on the subcellular morphology of activated platelets. Human (**a**–**c**) as well as wild-type (WT) and CypD^−/−^ (KO) murine platelets (**d**–**g**) were activated by thrombin+convulxin in the presence or absence of ROS and examined with TEM. For each treatment regimen, 10–25 images were analysed. The cumulative area (*TO*) and number (*n*) of membrane-bearing organelles, as well as the total platelet cross-section area (*TC*) were measured and the normalised organelle area was calculated as *TO/(n* × *TC)* and interpreted as an indicator of individual organelle swelling (**h**). The typical key changes of the organelle area are exemplified by the size and shape of the membrane-bearing organelles of panels (a) and (b). Mean values and SE are shown, asterisk represents a difference significant at P < 0.05 according to a post-hoc Tukey test performed after ANOVA (separately for human and murine datasets). *a: P = 0.0003 *b: P = 0.0038. Scale bar = 500 nm.

These qualitative observations warranted further structural investigations. Because of the known influence of CypD and MPTP on cellular integrity^9^, TEM imaging was performed to address the effect of CypD and CsA on the intracellular features of platelet activation. This technique requires that cells are activated in a suspension, therefore the collagen surface was replaced by its peptide analogue, convulxin. For quantitative assessment of the ultrastructural rearrangements a parameter independent of inherent variances in platelet cross-sections was used: the normalised organelle area, which was defined as the average area of membrane-bearing organelles per unit platelet cross section area in the TEM micrographs. ROS induced pronounced swelling of organelles in thrombin+convulxin-treated human platelets (Fig. 2a,b) as reflected in the 5-fold increase of the normalised organelle area (Fig. 2h), which could be completely reversed by CsA treatment (Fig. 2c,h). Genetic ablation of CypD also caused a slight decline in the normalised organelle area of murine platelets, however, this tendency failed to reach statistical significance regardless of the presence or absence of ROS (Fig. 2h). On the other hand, disruption of membrane integrity —which was more frequently seen in murine than in human ROS-treated samples— was largely abolished in CypD^−/−^ platelets (compare Fig. 2f and g).

### CypD and CsA effects on the fibrin structure formed in the presence of platelets activated by ‘strong’ stimuli

**Figure 3:**
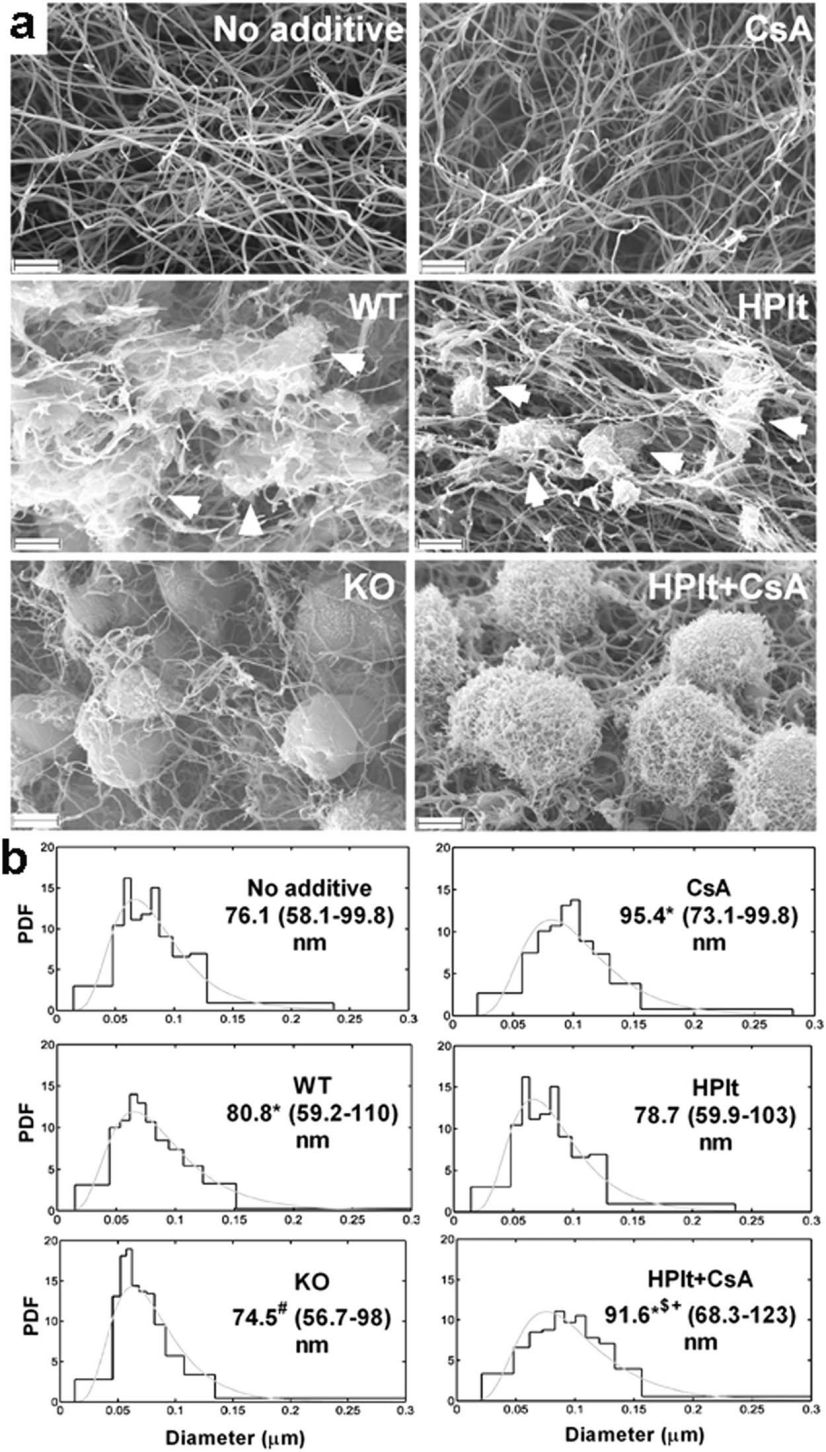
Platelet morphology in fibrin matrix. (**a**) Representative SEM images of fibrin clots prepared from 6 µM fibrinogen clotted for 2 h with 15 nM thrombin containing —where indicated— murine (WT: wild type or KO: CypD^−/−^) or human (HPlt) platelets at 0.5** × **10^5^/µl. In all cases 250 ng/ml convulxin was present in the clots. Where indicated, 1 µM CsA was also added. Scale bar = 2 µm. Arrowheads point to platelet fragments (WT and HPlt panels), in the vicinity of which bundles of fibrin fibres are assembled. This phenomenon is not observed around CypD^−/−^ and CsA-treated platelets (KO and HPlt + CsA panels). (**b**) The diameter of 300 fibres per image was measured from 2–6 SEM images per clot type using the algorithms described in Methods. The graphs present the probability density function (PDF) of the empiric distribution (histogram) and the fitted theoretical distribution (gray curves). The numbers under the clot type show the median, as well as the bottom and the top quartile values (in brackets) of the fitted theoretical distributions. Differences significant at P < 0.05 level according to Kuiper’s test are indicated with the following symbols: *(P < 0.001) compared to ‘No additive’; ^#^(P < 0.001) compared to ‘WT’; ^$^(P = 0.00249) compared to ‘CsA’; ^+^(P < 0.001) compared to ‘HPlt’.

In the course of clot formation, stimulated platelets are not only passively entrapped in the fibrin network, but they also modify the clot structure. Therefore, in further studies ‘strong’ stimuli were applied on platelets recruited to a three-dimensional fibrin matrix. SEM investigation of composite platelet-fibrin clots revealed that the combined stimulus of thrombin and convulxin caused the appearance of fragmented platelets (Fig. 3a, arrowheads) which were surrounded by thicker clusters of fibrin fibres. In contrast, platelets appeared to be more intact in clots containing CypD^−/−^ murine or CsA-treated human platelets with less heterogeneity in the surrounding fibrin fibres (e.g. WT vs. KO in Fig. 3a). Quantification of fibrin fibre diameters confirmed that incorporation of activated murine platelets caused a moderate thickening of fibrin fibres (the median diameter increased from 76.1 to 80.8 nm), while genetic ablation of CypD abolished this effect and reduced the heterogeneity of fibre size (Fig. 3b, left panels). Human platelets activated by thrombin+convulxin in the presence or absence of CsA had minor influence on fibre diameters when compared with appropriate controls to account for vehicle effects (‘HPlt’ vs. ‘No additive’ and ‘HPlt+CsA’ vs. ‘CsA’).

### CypD and CsA effects on platelet function as a response to ‘mild’ stimuli

While platelets in the core region of thrombi are likely to be exposed to ‘strong’ activators, ‘mild’ stimuli, such as ADP play a key role in platelet activation in the peripheral zones. To investigate the effect of CsA and CypD on this process, we carried out functional assays focusing on ADP-induced platelet adhesion, spreading and aggregation.

**Figure 4:**
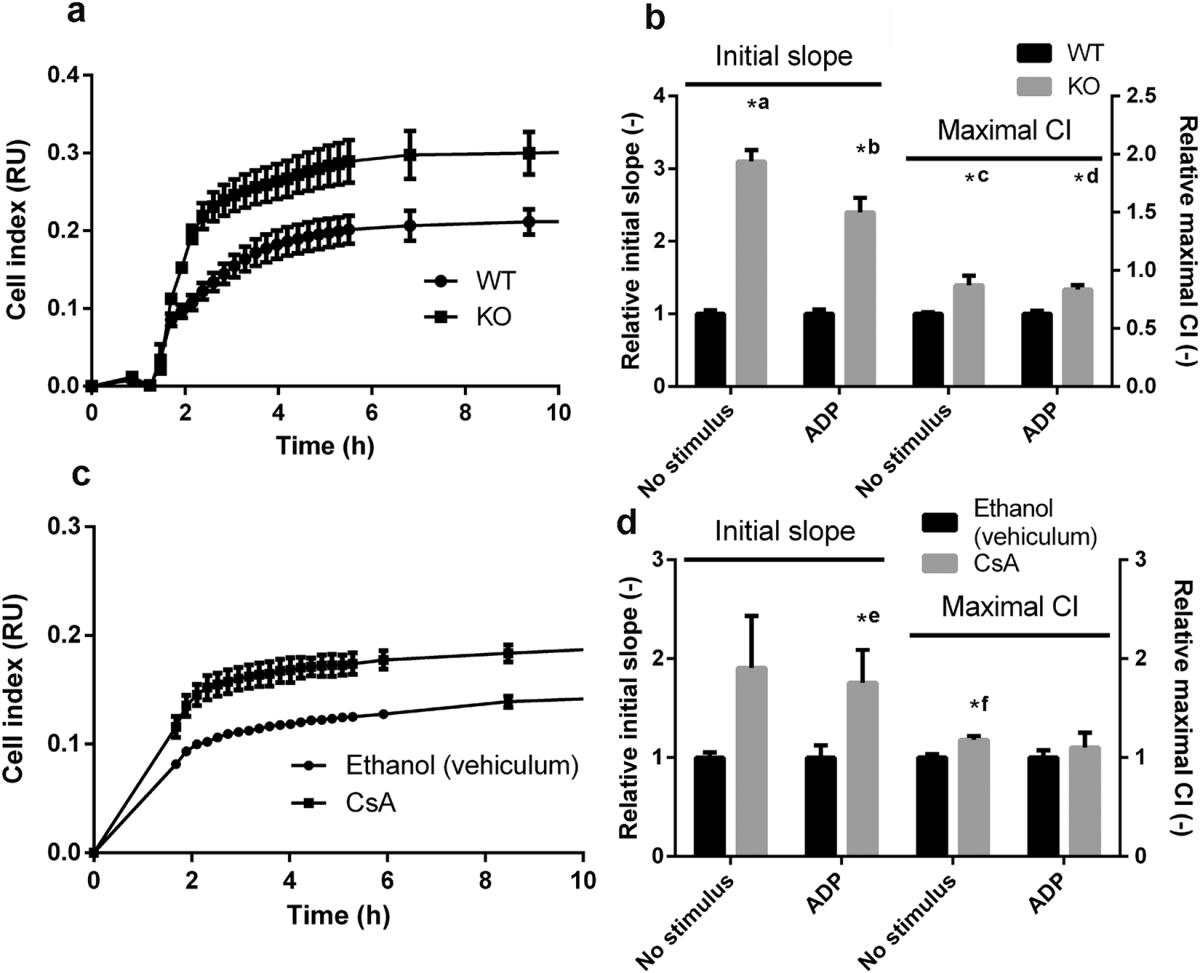
Adhesion and spreading of platelets measured by impedimetry. Impedance changes during adhesion and spreading of murine (**a**,**b**) and human (**c**,**d**) platelets. The change in impedance is represented as Cell Index (CI), a relative and dimensionless value which is calculated as CI = (Z_i_ − Z_0_)/F where Z_i_ is the impedance at an individual time point, Z_0_ is the impedance at the start of the experiment and F is a constant depending on the applied frequency. (**a**,**c**) Impedimetry traces: representative mean curves from 2–3 replicas. Bars represent SE. (**b**,**d**) Steepness and maximum Cell Index (CI) value were calculated from the impedimetry traces and are shown in relative units (the mean values of the steepness and maximum CI from the replicas of the vehicle control for human platelets and WT results for murine platelets were considered to be 1 for each independent set of measurements). Data are presented as means of n = 5–6, bars represent SE. Asterisks mark differences significant at P < 0.05 between the respective data pairs according to the Kolmogorov-Smirnov test. *a: P = 0.0013 *b: P = 0.0013 *c: P = 0.0013 *d: P = 0.0122 *e: P = 0.0183 *f: P = 0.0122. Abbreviations: WT, wild-type; KO, CypD^−/−^.

To examine real-time platelet-surface interactions, we adopted an impedance-based method described in recent studies^30,31^. Cells were allowed to adhere to, and spread on electrodes placed at the bottom of impedimetric wells and the change of impedance on the electrodes was measured. The initial, linear phase of the cell index (CI) curves reflect adhesion, while the maximal CI value corresponds to the area occupied by spreading platelets^31^. Genetic ablation of CypD resulted in an increased slope of impedimetry curves (by 210 and 140% without and with ADP, respectively) and in an increased maximum CI (by 40 and 34% without and with ADP, respectively) when compared to wild type (WT) platelets (Fig. 4a,b), reflecting increased adhesion and spreading of CypD^−/−^ platelets. Similar trends were observed with human platelets: CsA-treatment resulted in increased steepness (by 91 and 76%, without and with ADP, respectively) and maximum CI (by 18 and 10%, without and with ADP, respectively) of impedimetry curves (Fig. 4c,d).

**Figure 5:**
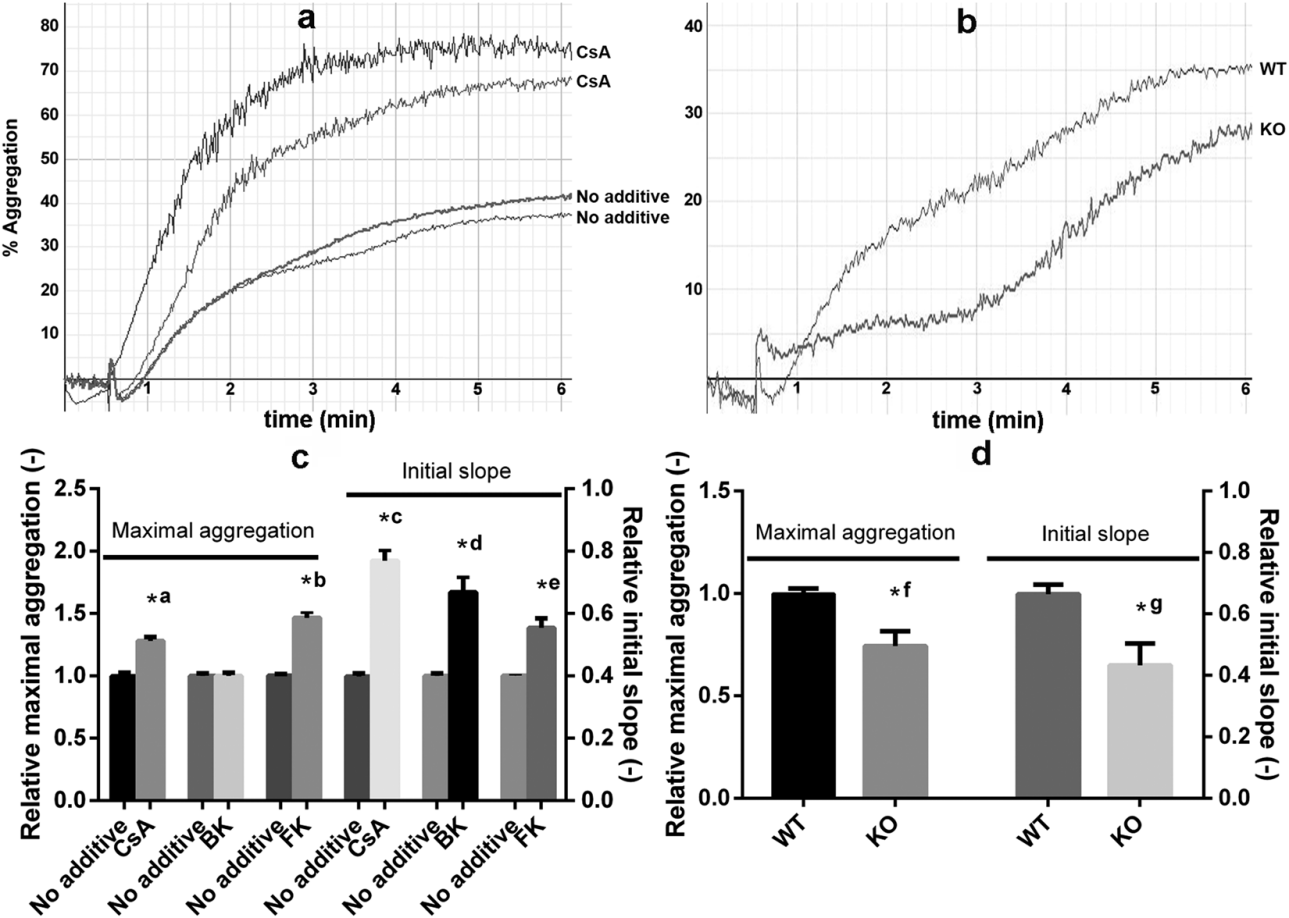
ADP-induced platelet aggregation. Washed human platelets were incubated with 2 µM CsA, 20 µM bongkrekic acid (BK) or 10 µM FK-506 (FK), or their respective vehicles (1% ethanol for CsA, 2 mM NH_4_OH for bongkrekic acid and 0.5% DMSO for FK-506) for 30 min at 37 °C. The conditioned human and the washed murine (wild-type: WT or CypD^−/−^: KO) platelets were supplemented with 6 µM fibrinogen to support aggregation right before it was initiated by the addition of 10–50 µM ADP (to give a maximal aggregation value of at least 30%) and 2.5 mM CaCl_2_. PBS was used as background for 100% transparency, and aggregation was recorded for 30 min. Aggregation curves from representative experiments using human (**a**) or murine (**b**) platelets are shown. (**c**,**d**) The measured initial slope and maximal aggregation values are shown in relative units (the mean value of the replicates of the vehicle control in each independent set of measurement was considered to be 1). ‘No additive’ designates samples incubated with the respective vehiculum of the inhibitors. Data from duplicates from at least three independent experiments and SE are shown. Asterisks mark differences significant at P < 0.05 between the indicated pairs of samples according to the Kolmogorov-Smirnov test. *a: P = 0.0438, *b: P = 0.0111, *c: P = 0.0026, *d: P = 0.0361, *e: P = 0.0111, *f: P = 0.0275 *g: P = 0.0042.

Platelet-platelet interactions were tested in an ADP-induced aggregometry assay. In the presence of CsA the initial slope of aggregation curves was almost doubled (1.00 ± 0.08 for control, 1.92 ± 0.26 for CsA) and the maximal aggregation values were also moderately increased (1.28 ± 0.09 with CsA compared to 1.00 ± 0.09) reflecting increased aggregation (Fig. 5a,c). In contrast, absence of CypD in murine platelets resulted in a decreased aggregation response to ADP (Fig. 5b,d) as initial slope and maximal aggregation values were lower by 35 and 26%, respectively. To clarify the discrepancy between CsA an CypD effects, selective modifiers of calcineurin and mitochondrial energetics were tested in this assay. Bongkrekic acid [Bk, an inhibitor of adenine nucleotide translocator (ANT)] treatment resulted in a pronounced (67%) increase in initial slope values, while maximal aggregation remained essentially unchanged. FK-506 (a selective inhibitor of calcineurin) in the 100–1000 nM range had no effect on aggregation parameters (results not shown), while higher concentrations (5–50 µM) resulted in increased maximal aggregation values (1.47 ± 0.1 with FK-506 compared to 1.00 ± 0.03) (Fig. 5c).

### Modification of the fibrin structure formed in the presence of platelets activated by ‘mild’ stimuli

To test how ‘mild’ activation of platelets affects the surrounding fibrin structure, platelets pre-treated with various modulators were activated by ADP (Table 1). Pre-treatment of human and WT murine platelets with CsA or Bk resulted in thinner fibres, whereas using CypD^−/−^ platelets Bk-treatment decreased the fibre diameter, but CsA-treatment had no effect. Incorporation of FK-506-treated human and murine platelets resulted in a decrease in the median fibre diameter from 84.9 nm to 78.5 nm with human platelets and an increase with murine platelets (from 85.9 nm to 92.3 nm in the presence of WT and from 91.3 nm to 109.4 nm with CypD^−/−^ platelets). The addition of ROS did not alter these tendencies, although it generally increased the diameter of fibrin fibres, e.g. in clots containing CypD^−/−^ platelets median values were 17–27 nm higher in the presence of ROS than in their absence (compare data for inhibitor-free vehicle samples in Table 1, all P < 0.0001).

**Table 1:** Effects of modulators of platelet function on the structure of fibrin formed in the presence of human and murine platelets.

Platelet	cyclosporine A			FK-506			bongkrekic acid		
	—	+	P	—	+	P	—	+	P
Human	94.2 [75.0–118.2]	86.8 [66.9–112.7]	0.0017	84.9 [67.9–106.2]	78.5 [63.4–97.3]	0.0042	86.2 [69.8–106.4]	78.4 [64.4–95.4]	0.0008
WT	97.8 [80.1–119.4]	85.9 [70.4–104.7]	0.0000	85.9 [70.0–105.3]	92.3 [74.5–114.4]	0.0067	99.6 [79.8–124.3]	90.6 [73.4–112.0]	0.0008
CypD^−/−^	96.9 [74.6–125.9]	98.5 [76.4–126.8]	0.7630	91.3 [70.8–117.7]	109.4 [85.3–140.2]	0.0000	107.6 [83.0–139.5]	100.4 [80.2–125.7]	0.0017
Human+ROS	97.6 [79.4–119.9]	91.3 [72.7–114.8]	0.0133	109.6 [86.6–138.7]	87.2 [67.7–112.3]	0.0000	96.2 [74.2–124.7]	89.7 [71.5–112.5]	0.0075
WT+ROS	97.6 [77.0–123.7]	88.2 [69.9–111.5]	0.0008	90.8 [71.9–114.8]	107.5 [85.8–134.8]	0.0000	111.8 [87.8–142.4]	98.6 [78.5–123.9]	0.0000
CypD^−/−^+ROS	115.0 [89.2–148.4]	110.0 [84.6–143.0]	0.2650	108.1 [86.3–135.4]	128.4 [98.3–167.7]	0.0000	134.7 [104.9–172.9]	97.1 [73.5–128.1]	0.0000

The table shows the median fibrin fibre diameters with lower and upper quartiles in brackets determined using the algorithm described in Methods from the measurement of diameter of 300 fibrin fibres per image from 2–6 scanning electron microscopic images per clot type. Fibrin was prepared from 6 µM fibrinogen clotted for 2 h with 15 nM thrombin in the presence of human or murine (WT: wild type or CypD^−/−^) platelets at 10^5^/µl. Where indicated (+) platelets were incubated with 2 µM cyclosporine A, 20 µM bongkrekic acid, 10 µM FK-506 or their respective vehicle (1% ethanol for CsA, 2 mM NH_4_OH for bongkrekic acid, 0.5% DMSO for FK-506) and 10 μM ADP for 30 min prior to clotting. The P-values refer to Kuiper’s statistical test for differences of distributions of the fibre diameters in the presence and absence of the respective inhibitor.

### Impact of modulators of platelet activation on the lytic susceptibility of fibrin

**Figure 6:**
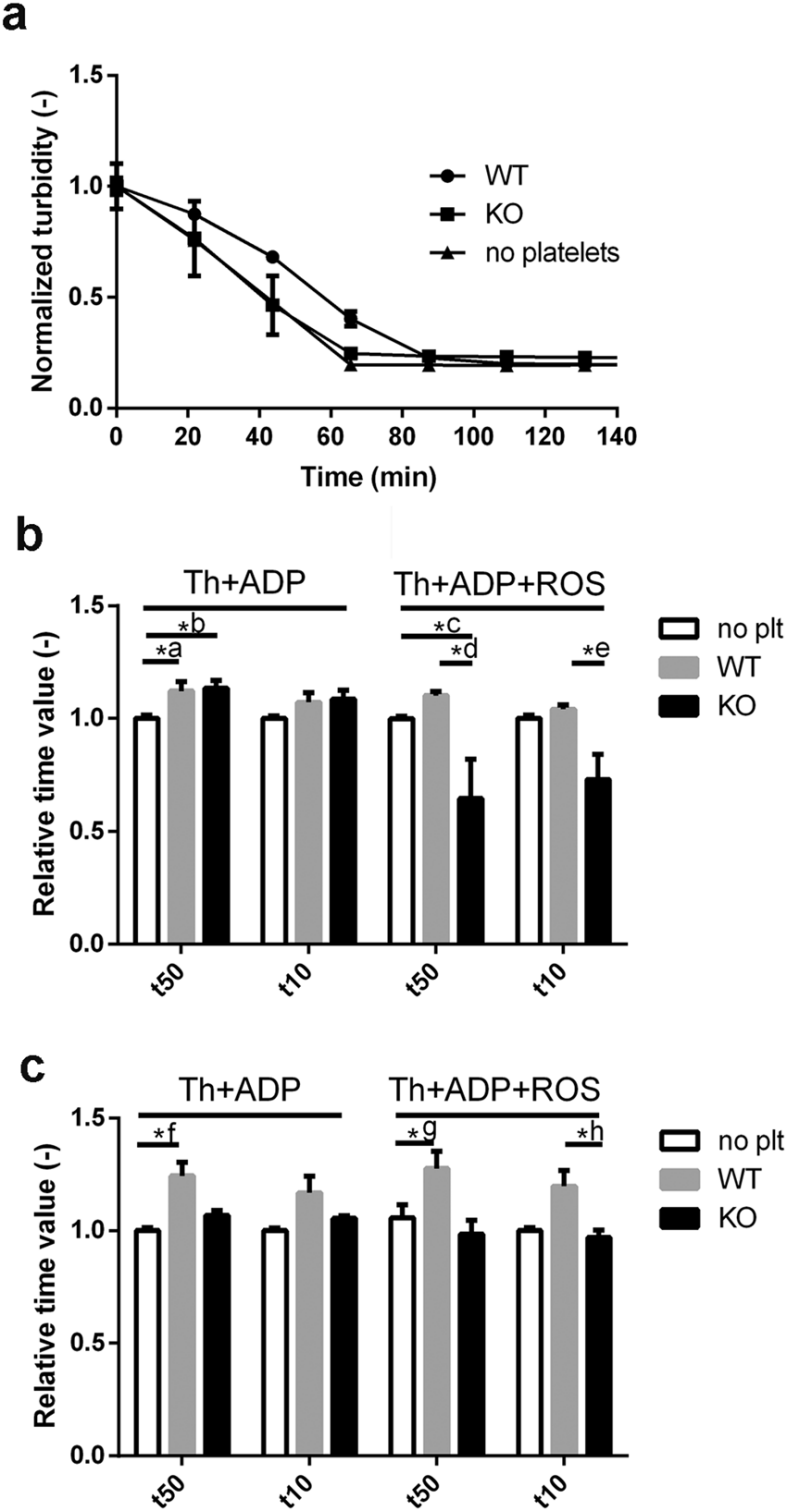
tPA-induced lysis of platelet-containing fibrin clots. Fibrinogen (6 µM) supplemented with 0.5 μM plasminogen, 2.5 mM CaCl_2_, 10 μM ADP and in the indicated cases, with murine platelets and modulator additives was clotted with 16 nM thrombin for 60 min. Lysis was initiated by addition of 50 µg/ml tPA to the clot surface and light absorbance at 340 nm was measured. (**a**) Representative lysis curves of tPA-induced lysis in clots containing ROS and platelets isolated from wild type (WT) and CypD^−/−^ (KO) mice. The presented turbidity values are the measured absorbance values normalised for the maximal absorbance for each sample (n = 4, every 10^th^ measurement point is shown, bars represent SE values). The time to reach 50% (t50) and 10% (t10) of the initial turbidity was calculated from the lysis curves of clots containing (**b**) 1** × **10^5^/µl or (**c**) 2** × **10^5^/µl platelets and presented in relative units: the mean value of the t50 and t10 for platelet-free fibrin (no plt) in each independent set of measurement was considered to be 1. Mean of n = 7–8 replicates and SE values are shown. Asterisks indicate significant differences at P < 0.05 according to the post-hoc Dunn test performed after Kruskal-Wallis test for the respective triplets of data. *a: P = 0.0241, *b: P = 0.0241, *c: P = 0.0154, *d: P = 0.0021, *e: P = 0.0034, *f: P = 0.0009 *g: P = 0.0184, *h: P = 0.0239.

At sites of their activation platelets interact with a heterogeneous fibrin network forming the scaffold of haemostatic clots and pathological thrombi. There is continuing therapeutic interest in the effective enzymatic dissolution of the fibrin network in several clinical settings. In view of the role of platelets in fibrin stabilisation (reviewed by Longstaff & Kolev^27^), we examined the effects of CsA and CypD on the fibrinolytic susceptibility of model thrombi. Modifying a global turbidity-based assay that has been successfully used in our earlier studies^32^, we incorporated platelets at different cell counts into a fibrin matrix containing various activators. (i) Thrombin played a dual role in this assay catalysing the fibrinogen-fibrin conversion and activating platelets. (ii) In certain cases ROS were added to represent oxidative agents released from dying cells and neutrophils in thrombi. (iii) The mild activator ADP was used to match the milieu of peripheral regions of thrombi, which are more susceptible to circulating fibrinolytic agents. Proteolytic digestion of the formed clot was initiated by the most clinically relevant fibrinolytic agent, tissue-type plasminogen activator (tPA) (Fig. 6a). In all experimental setups, the presence of WT murine platelets caused a delay in fibrinolysis (Fig. 6b,c). Remarkably, genetic ablation of CypD largely reversed this effect: the t10 or t50 values of fibrin containing platelets from CypD^−/−^ mice were shifted towards those of platelet-free fibrin. These tendencies were more evident at higher platelet counts (compare left side of Fig. 6b and c), and the differences were most pronounced in the presence of ROS (right side of Fig. 6b and c). CsA treatment had no effect on the progress of lysis curves in fibrin clot models containing human platelets (data not shown).

## Discussion

Previous studies on the role of MPTP in platelet activation have suggested CypD as an attractive target to influence thrombus formation^5,10,20^. However, the findings of these studies are controversial, possibly stemming from the spatiotemporal heterogeneity of platelet subpopulations in growing thrombi^33^. In a photochemical injury model, genetic ablation of CypD accelerated thrombosis^5^, and this was confirmed with megakaryocyte/platelet-specific CypD-knockout platelets in a mesenteric arterial thrombosis model^20^. In contrast, deletion of CypD hampered thrombus formation in a study using FeCl_3_ injury^10^. These controversial results from studies using global *in vivo* models as well as the thrombotic complications of patients treated with CsA warranted further *ex vivo* investigation of CypD and CsA effects under specific conditions. Accordingly, with the present study we addressed the consequences of the genetic deletion of CypD and its acute inhibition by pharmacological agents in terms of distinct platelet functions. We found that CypD-deficiency increased spreading (Fig. 4) and impaired aggregation (Fig. 5) of murine platelets in response to a ‘mild’ stimulus (ADP); and accelerated the resolution of fibrin-platelet clots formed in the presence of ‘mild’ and ‘strong’ stimuli (Fig. 6). The effects of pharmacological inhibition of CypD in human platelets generally followed the same trends, however, important differences will also be discussed.

MPTP opening is characterised by the loss of ion gradients across the inner mitochondrial membrane, organelle swelling, and eventually, disruption of plasma membrane integrity^9^. Platelets activated by ‘strong’ stimuli that induce MPTP formation share these characteristics, and have been recently termed ‘necrotic’^10^. An earlier study^5^ showed that membrane integrity of fibrin-adherent murine platelets challenged with ‘strong’ activators can be restored by genetic ablation of CypD. Our SEM studies have expanded this observation using collagen-adherent as well as fibrin-entrapped platelets. Moreover, we report that CsA treatment of human platelets is able to reproduce the ultrastructural effects of CypD deletion under these circumstances. To detect and quantify another ultrastructural characteristic of MPTP opening (organelle swelling), we utilised TEM. To our knowledge, the aforementioned study^5^ has been the only one in this field so far in which TEM investigations were undertaken, however, the authors do not show any micrographs or quantification. According to the evaluation of our TEM images, CsA effectively counteracted ROS effects in terms of organelle swelling in human platelets. Our and others’^5^ failure to detect quantifiable differences in subcellular structures of activated WT and CypD^−/−^ platelets might be explained by chronic metabolic alterations resulting in rupture of membrane-coated vesicles. In our study, only organelles surrounded by membranes were measured, therefore excessively swollen vesicles that lost their membrane integrity were not included in the analysis. Alternatively, the discrepancies between the effects of CypD ablation and CsA effects might underline the importance of CypD-independent CsA effects that protect mitochondrial and cellular integrity^34^.

Adhesion and aggregation of platelets are of fundamental importance to prevent blood loss. Platelets are known to adhere to a variety of surfaces (collagen, fibrin, gold^35^) and go through consequential shape changes (flattening known as ‘spreading’). Previous studies have reported increased spreading in the absence of CypD^5,20^. However, these studies were carried out using ‘strong’ stimuli, and the methods used (SEM^5^, fluorescent microscopy^20^) do not provide information on adhesion. The impedance-based method enabled us to follow real-time adhesion *and* spreading dynamics in a single assay, and we found that both aspects of platelet activation were enhanced not only in the absence of CypD, but also during its inhibition by CsA in the presence of the ‘mild’ activator ADP. An apparent limitation of this experimental setup is the lack of physiological coating on electrodes. However, it is important to note that (i) for the purposes of our investigation, the isolated assessment of ‘mild’ stimulus (ADP) effects necessitated the absence of proteins that might interfere with platelet activation (e.g. collagen or fibrinogen); (ii) our impedimetric curves reach maximum CI values almost identical to those reported in other studies^30^, where the authors used collagen coating [although the stimuli are naturally different, the platelet counts are very similar in the two studies (10^5^/µl versus 1.25 × 10^5^/µl)].

Regarding platelet-platelet interactions, CsA has been long shown to increase aggregation *in vitro*^36–38^, in line with our current data. On the other hand, previous data concerning the effects of CypD-deletion on ‘strong’-stimulus-induced aggregation are not consistent. CypD-deletion either had no effect^5^ or enhanced aggregation^20^, while other authors have proposed that the change in light transmission measured in aggregometry assays in response to thrombin + convulxin reflected a shape change of platelets rather than aggregate formation^21^. However, to our knowledge, the current study is the first to test aggregation of CypD^−/−^ platelets in response to ADP, a ‘mild’ trigger acting independently of MPTP formation. Here we report that ADP-activated platelets of CypD^−/−^ mice exhibited lower aggregation propensity compared to WT platelets. We tested two hypotheses that might account for the disparate aggregation patterns with CsA-inhibition and CypD-deletion in our study. (i) CypD-deletion might impair aggregation through alteration of the metabolic status of platelets. It is known that ablation of CypD causes substantial changes in the levels of numerous mitochondrial enzymes involved in intermediary metabolism^39^. Such an altered metabolic status might lead to impaired aggregation since this response is of relatively high metabolic cost among ADP-induced activation events^40^. As a crude model of this scenario, we used Bk, an inhibitor of mitochondrial ADP-ATP exchange^41^. It is important to note that the Bk-target ANT is a well-recognized modulator of MPTP^42^. However, in our aggregation assay, where MPTP formation is absent, the effect of Bk on the initial slope of aggregation could be attributed to alteration of the cellular metabolic status by interference with the mitochondrial ADP-ATP exchange. Nevertheless, Bk treatment of human platelets failed to reproduce the impaired aggregation response of CypD^−/−^ platelets, suggesting a significant role for the long-term metabolic adaptation of CypD^−/−^ platelets in their modified functional response (rather than for the acute impairment of ATP generation). (ii) CypA- and not CypD-mediated effects of CsA might dominate the aggregation response seen with human platelets. It is known that CsA binds to both intra- and extracellular forms of CypA. However, given that extracellular CypA secreted during platelet activation promotes aggregation and adhesion^43^, this pathway is likely to have negligible effect in our and others’^36–38^ experimental systems where CsA-treatment enhanced aggregation and adhesion responses —in line with the thrombotic complications observed in patients undergoing CsA-treatment^26^. Therefore, we focused on the effects of CsA on the intracellular form of CypA resulting in inhibition of calcineurin. Despite earlier, variable results with calcineurin-inhibition in platelets^37,38^, recently, deletion of an isoform of the catalytic subunit of calcineurin as well as FK-506-treatment have been shown to increase ADP-triggered aggregation^25^. In line with this finding, FK-506 and CsA had similar effects in our aggregation assay. Taken together, these results suggest that the positive effect of CsA in our mild stimulus-induced aggregation studies is primarily not exerted through CypD, rather via binding to CypA and consequent inhibition of calcineurin.

During thrombus formation, platelets play a major role in shaping the characteristics of the fibrin scaffold. This effect is based on various mechanisms: modulation of thrombin concentration through provision of pro-coagulant phospholipid surface, mechanical retraction caused by platelet contraction, promotion of the catalytic activity of factor XIII (as reviewed by Longstaff & Kolev^27^). Our coagulometric assays (see Supplementary data) clearly showed that the pro-coagulant effect of platelets in the tissue-factor-induced clotting of plasma was not affected by the modulators of platelet function applied in our study (CypD-ablation, CsA-, FK-506- or Bk-treatment). Thus, excluding this mechanism, we restricted our evaluation of platelet effects on fibrin structure to a purified system using isolated platelets and thrombin-induced fibrin formation, in which Ca^2+^-dependent platelet contraction and factor XIII crosslinking are key determinants of fibrin structure. In clots containing human platelets both CsA and FK-506 resulted in a decreased fibrin fibre diameter consistent with a role for calcineurin signalling in the CsA effects. However, CypD inhibition was also partially accountable for the effects of CsA in this assay, based on the finding that CsA decreased the fibrin diameter in the presence of WT, but not CypD^−/−^ murine platelets. An interesting unexpected finding also supported the primary role of CypD inhibition in the mechanism of CsA action: despite the thinning effect of CsA, selective calcineurin inhibition with FK-506 resulted in thicker fibres in the presence of both WT and CypD^−/−^ platelets. These disparate effects of FK-506 in platelets of human and murine origin indicate a species-dependent difference in the role of calcineurin in platelet activation. A plausible source of this difference might be the thrombin-sensitizing effect of protease-activated receptor (PAR) 3 on PAR4 activation in mice, an effect absent in human platelets^44,45^.

The therapeutic modality of thrombolysis relies on administration of agents (e.g. tPA) that promote digestion of the fibrin network (fibrinolysis) resulting in the disassembly of the clot. Thrombolysis is the first-line treatment in ischemic stroke^46^, an option in certain cases of deep vein thrombosis^47^, as well as for selected patients with acute myocardial infarction^48^. However, this therapeutic approach often fails and/or causes bleeding as a side effect^49^, which warrants further studies on the determinants of thrombolytic efficacy. As essential constituents of haemostatic plugs and pathological thrombi, platelets are known to render clots resistant to physiological as well as therapeutic fibrinolysis through a variety of mechanisms. In addition to the structural modifications in fibrin discussed above, these mechanisms include secretion of plasminogen activator inhibitor PAI-1 and α_2_-plasmin inhibitor, release of phospholipids and myosin (as reviewed by Longstaff & Kolev^27^). We report that while incorporation of WT murine platelets in fibrin clots resulted in a delay in tPA-induced fibrinolysis, absence of CypD largely abolished this effect, specifically under conditions of oxidative stress. Previously, increased retraction was found in plasma clots containing CypD^−/−^ platelets^5^, which suggests lytic resistance. However, our study has identified additional factors that can reverse the anti-fibrinolytic effects of enhanced clot retraction in the absence of CypD. (i) Electron microscopy showed preserved ultrastructural integrity of CypD^−/−^ platelets, implying limited release of anti-fibrinolytic constituents (e.g. phospholipids, myosin). (ii) The presence of CypD^−/−^ platelets resulted in larger fibrin fibre diameters compared to WT platelets with a stronger effect under oxidative stress (compare the diameters in the inhibitor-free clots for the respective WT/CypD^−/−^ pairs in Table 1). Since fibre diameter is an important determinant of tPA-induced lysis (coarse meshwork with thicker fibres being more susceptible)^50^, this finding might also contribute to the observed differences in tPA-induced degradation of clots containing WT and CypD^−/−^ platelets.

In summary, our findings —taken together with other published data— suggest that caution is necessary if therapeutic benefit is sought through modulation of CypD-dependent pathways in platelets. Firstly, the outcome of CypD targeting is dependent on the stimulus of platelet activation (e.g. different effects of CypD ablation with ‘strong’ vs. ‘mild’ stimulus-induced aggregation). Secondly, the net impact of CypD inhibition on thrombosis is difficult to predict as it is likely to be a result of opposing (pro-thrombotic and pro-fibrinolytic) effects (e.g. increased adhesion of platelets versus decreased fibrinolytic resistance of clots in the absence of CypD). Finally, CsA-treatment of human platelets does not fully reproduce the effects of CypD deletion. In view of the thrombotic complications associated with CsA therapy, these conclusions warrant further *in vitro* and *in vivo* research with selective inhibitors of CypD to detect which of the mechanisms identified in different studies can be translated into actual clinical benefit.

## Methods

### Animals

WT and CypD^−/−^ littermate mice (of C57Bl/6 J background, either sex, age between 60 and 180 days) were housed in a room maintained at 20–22 °C on a 12 h light–dark cycle with food and water available ad libitum. Experiments were approved by the Animal Care and Use Committee of Semmelweis University and were performed in accordance with the guidelines and regulations of the Code of Animal Welfare and Experimentation Rules of Semmelweis University (act #106/b//2013., IX. 26).

### Preparation of platelets

Mice were anaesthetised by intraperitoneal injection of 400 mg/kg chloralhydrate. Terminal blood collection was performed through the inferior caval vein after median laparotomy. Blood samples were collected into 85 mM trisodium citrate, 66 mM citric acid, 80 mM glucose at an anticoagulant/blood ratio of 1:9. Citrated human blood was obtained from healthy individuals. Platelet rich plasma (PRP) was prepared by centrifugation of samples at 150 *g* for 10 min at 25 °C. PRP was diluted 4-fold in 63 mM TRIS, 95 mM NaCl, 12 mM citric acid, 1 µM PGE_1_, 1 U/ml apyrase, pH 6.5 and centrifuged at 600 *g* for 10 min at 25 °C. The supernatant was discarded, and platelets were resuspended in PBS (phosphate buffered saline: 134 mM NaCl, 2.9 mM KCl, 20 mM Na_2_HPO_4_, 12 mM NaHCO_3_, 1 mM MgCl_2_, 5 mM glucose, pH 7.35). Cell counts were determined using an Abacus Junior B Hematology Analyser (Diatron, Budapest, Hungary).

### Effect of platelets on tissue factor induced clotting of plasma

Washed platelets (at a final count of 10^5^/µl) were incubated with 2 µM CsA, 50 µM FK-506 or 20 µM Bk or their vehicles (1% ethanol, 0.5% DMSO or 2 mM NH_4_OH, respectively) for 30 min at 37 °C. Following pre-activation with 10 µM ADP, platelet suspensions were mixed with citrated human pooled plasma at a ratio of 1:1 and supplemented with 12.5 mM CaCl_2_. Clotting was initiated by the addition of 1200 × diluted recombinant thromboplastin Dia-PT R (Diagon Kft, Budapest, Hungary) and monitored in two assay settings. (i) Clotting time measurement in a coagulometer KC-1A (Amelung, Lemgo, Germany) at 37 °C. (ii) Turbidimetric assay, in which clot formation was monitored by registering the absorbance at 340 nm at 37 °C with a CLARIOstar microplate reader (BMG LABTECH, Ortenberg, Germany) and clotting time defined as time to reach half-maximal absorbance.

### Scanning electron microscopic imaging

Washed platelets at 1.5 × 10^5^/µl in PBS containing 2.5 mM CaCl_2_ were placed on glass cover slips coated with 0.05 mg/ml collagen G (Biochrom AG, Berlin, Germany) in 150 mM NaCl 10 mM HEPES pH 7.4 overnight at 4 °C. Platelets were activated with 25 nM thrombin (Serva Electrophoresis GmbH, Heidelberg, Germany, further purified and characterized as described previously^51,52^ for 0/15/30 min at 37 °C in the absence or presence of 2 µM CsA. In certain cases, 10 µM CuSO_4_, 10 µM homocysteine and 100 µM H_2_O_2_ (indicated as ROS whenever applied in the measurements) were also added. This ROS mix was selected to generate hydroxyl radicals through a copper/thiol-dependent chemistry^28,29^ at physiologically relevant concentrations of the components. Hydroxyl radical formation was confirmed with the fluorescent probe coumarin-3-carboxylic acid^53^. For the preparation of fibrin clots, 6 µM fibrinogen (human, plasminogen depleted from Calbiochem, LaJolla, CA, USA) was supplemented with washed platelets at 0.5 × 10^5^/µl in PBS containing 2.5 mM CaCl_2_ and 250 ng/ml convulxin ± 1 µM CsA. In another experimental setup washed platelets were pretreated with 2 µM CsA/50 µM FK-506/20 µM Bk or their respective vehicles for 30 min at 37 °C. Fibrin clots were prepared by mixing 6 µM fibrinogen with platelets at 10^5^/µl, 2.5 mM CaCl_2_ and 10 µM ADP in the presence or absence of the previously described ROS mix. The samples were clotted with 15 nM thrombin for 2 h. Platelet activation and clot formation were terminated using 3% glutaraldehyde in 100 mM Na-cacodylate pH 7.2. Further treatment of samples as well as generation and analysis of images was carried out as described previously^50^.

### Transmission electron microscopic imaging

Washed platelets at 3.3 × 10^5^/µl in PBS containing 2.5 mM CaCl_2_ were activated with 25 nM thrombin and 250 ng/mL convulxin at 37 °C. In certain cases, ROS ± 2 µM CsA were also added. After 2 h of activation, platelets were centrifuged at 1500 *g* for 5 min. Supernatants were discarded, samples were treated with 30 µl 3% glutaraldehyde in 100 mM Na-cacodylate pH 7.2 for 16 hours, followed by centrifugation as described above. The pellet was treated with 1% OsO_4_ (in distilled water) for 6 hours at 4 °C. After washing with distilled water, the cells were suspended in 10 µl of 1% low melting-point agarose at 40 °C. After congelation, 1 mm^3^ pieces were dehydrated in 20–96% (v/v), absolute ethanol, propylene oxide, then infiltrated and embedded in Durcupan ACM Epoxy Resin. Resin blocks were polymerised for 48 h at 60 °C and 80 nm sections were made using Leica UC7 ultramicrotome (Leica Microsystems, Wetzlar, Germany). Sections were mounted on 100-mesh nickel grids coated with Formvar (SPI-Chem, West Chester, PA, USA), contrasted with 1% (w/v) aqueous uranyl acetate and 0.08% (w/v) lead citrate, and examined using a Philips Morgagni 268D electron microscope (FEI, Eindhoven, Netherlands) at an accelerating voltage of 100 kV. Images were analysed using siViewer 5.1 (Olympus Soft Imaging Solutions, Münster, Germany) and ImageJ (NIH, Bethesda, MD) software. From TEM images depicting individual platelet cross-sections, total area of membrane-bearing organelles (TO) as well as total platelet cross-section area was measured (TC). Normalised organelle area was calculated as TO/(n × TC) for each evaluated image, where n is the number of detected organelles.

### Investigation of platelet adhesion and spreading

Cell adhesion and surface spreading were monitored in E-plates (ACEA Biosciences, Ind., USA) in an xCELLigence System (Roche Applied Science, Indianapolis, USA)^30,31^. During the attachment of cells an increase in the impedance can be registered, which depends on the local ionic environment, the number and spreading of cells adhered to the surface of the electrodes at the bottom of wells. The change in impedance is represented as Cell Index (CI), which is calculated as CI = (Z_i_ − Z_0_)/F where Z_i_ is the impedance at an individual time point, Z_0_ is the impedance at the start of the experiment and F is a constant depending on the applied frequency.

PBS was added to each well and the impedance was detected for 1 h to obtain constant background values. Thereafter, 2.5 mM CaCl_2_ ± 10 µM ADP were added and a second baseline was measured. After addition of platelets at 10^5^/µl, the impedance was recorded at 10 kHz for 24 h, using only PBS-containing wells as control. [Human platelets at 2 × 10^5^/µl in PBS were pre-incubated with 2 µM CsA or 1% ethanol (as vehicle control) for 30 min at 37 °C]. Impedance curves were analysed using the Curve fitting toolbox of Matlab 7.10.0.499 (R2010a) (The Mathworks, Natick, MA, USA) software to calculate maximal impedance and initial slope values.

### Measurement of platelet aggregation

Aggregation was monitored with a Carat-TX4 optical aggregometer (Carat Diagnostics, Budapest, Hungary)^54^. Washed platelet count was adjusted to 2 × 10^5^/µl by dilution with PBS containing 2.5 mM CaCl_2_ and 6 µM fibrinogen, and aggregation was induced by 10–50 µM ADP after 30 min of incubation with CsA, Bk or FK-506, or their vehicles. Aggregation was expressed as percentage of maximal transparency measured with PBS. Aggregation curves were analysed with the integrated software of the instrument to calculate initial slope and maximal aggregation values.

### Fibrinolysis assays

tPA-driven lysis was studied in clots prepared from 6 µM fibrinogen supplemented with 0.5 µM plasminogen (isolated from human plasma^55^), 2.5 mM CaCl_2_, human or murine platelets at 0/1/2 × 10^5^/µL, and clotted with 16 nM thrombin, in a total volume of 120 µl. Lysis was initiated by addition of 100 µl of 50 µg/mL tPA to the clot surface. Clot formation and dissolution was followed by measuring the light absorbance at 340 nm at 37 °C with a Zenyth 200rt microplate spectrophotometer (Anthos Labtec Instruments GmbH, Salzburg, Austria). Lysis curves were analysed with a self-designed script running under the Matlab software to determine the time needed to reduce the turbidity of the clot to a given fraction of the maximal (initial) value (t50 to reach 0.5A_max_, t10 to reach 0.1A_max_) as a quantitative parameter of fibrinolytic activity^32^.

### Statistical analysis

The distribution of fibrin fibre diameter data was analysed using the algorithm described previously^50^: theoretical distributions were fitted to the empirical data sets and compared using Kuiper’s test and Monte Carlo simulation procedures. On other datasets with three or more compared subsets, ANOVA was performed. For datasets with small sample sizes (n < 9), where normal distribution of the evaluated numeric data could not be confirmed, non-parametric statistical tests were applied. The Kolmogorov-Smirnov test was chosen because of its robust power to compare distributions of two data sets independently of their distribution. For the comparison of three or more datasets, the Kruskal-Wallis test was used. All statistical tests were performed using GraphPad Prism 6.00 (GraphPad Software, La Jolla California USA) and the Statistical Toolbox 7.3 of Matlab. Data are presented as mean ± standard error of mean (SE), except for fibrin fibre diameter given as median and bottom–top quartile values.

### Data availability

The datasets generated during and/or analysed during the current study are available from the corresponding author on reasonable request.

## Electronic supplementary material

Supplementary data
