# Supplementary material for: Functional cyclophilin D moderates platelet adhesion, but enhances the lytic resistance of fibrin
Source: Sci Rep. 2018 Mar 29;8:5366. doi: 10.1038/s41598-018-23725-4 (PMC5876378; doi:10.1038/s41598-018-23725-4)
Supplement: Supplementary file 1 — Supplementary data [file 41598_2018_23725_MOESM1_ESM.pdf]

Supplementary data

Functional cyclophilin D moderates platelet adhesion, but enhances the lytic resistance of fibrin

Imre Varjú,[1,2] Veronika Judit Farkas,[1] László Kőhidai,[3] László Szabó,[4] Ádám Zoltán Farkas,[1] Lívia Polgár,[3] Christos Chinopoulos[1,5] and Krasimir Kolev[1]*

[1]Department of Medical Biochemistry, Semmelweis University, Budapest, 1094, Hungary
[2]Program in Cellular and Molecular Medicine, Boston Children's Hospital, Harvard Medical School, Boston, 02115, USA
[3]Department of Genetics, Cell- and Immunobiology, Semmelweis University, Budapest, 1089, Hungary
[4]Department of Functional and Structural Materials, Institute of Materials and Environmental Chemistry, Research Centre for Natural Sciences, Hungarian Academy of Sciences, Budapest, 1117, Hungary
[5]MTA-SE Lendület Neurobiochemistry Research Group, Budapest, 1094, Hungary
*Krasimir.Kolev@eok.sote.hu

Effects of platelets on the tissue factor induced clotting of plasma

The pro-coagulant effects of platelets were examined in two experimental setups. Table S1 presents the tissue factor induced clotting times from the coagulometric assay described in the Methods section. Figure S1 shows the time to reach half-maximal absorbance (t50) in the turbidimetric assay described in the Methods section. Both assays indicate that the presence of platelets increases the rate of clot formation, however no differences were seen in relation to the applied modulators of platelet function.

Table S1. Effect of platelets on tissue factor induced clotting time. Clotting time was measured with a coagulometric assay. The table reports the mean±SD values of two independent experiments with three parallel measurements. PPP: human platelet-poor pooled plasma, Plt: platelet. Addition of ADP did not change the clotting time in the presence of platelets ($p>0.05$ for (PPP+Plt) v. (PPP+ADP+Plt), Kolmogorov-Smirnov statistical test).

	PPP	PPP+Plt	PPP+ADP+Plt
Clotting time (s)	99.13±1.59	93.47±0.45	93.4±0.7
	84.23±3.68	80.17±0.7	81.63±1.32

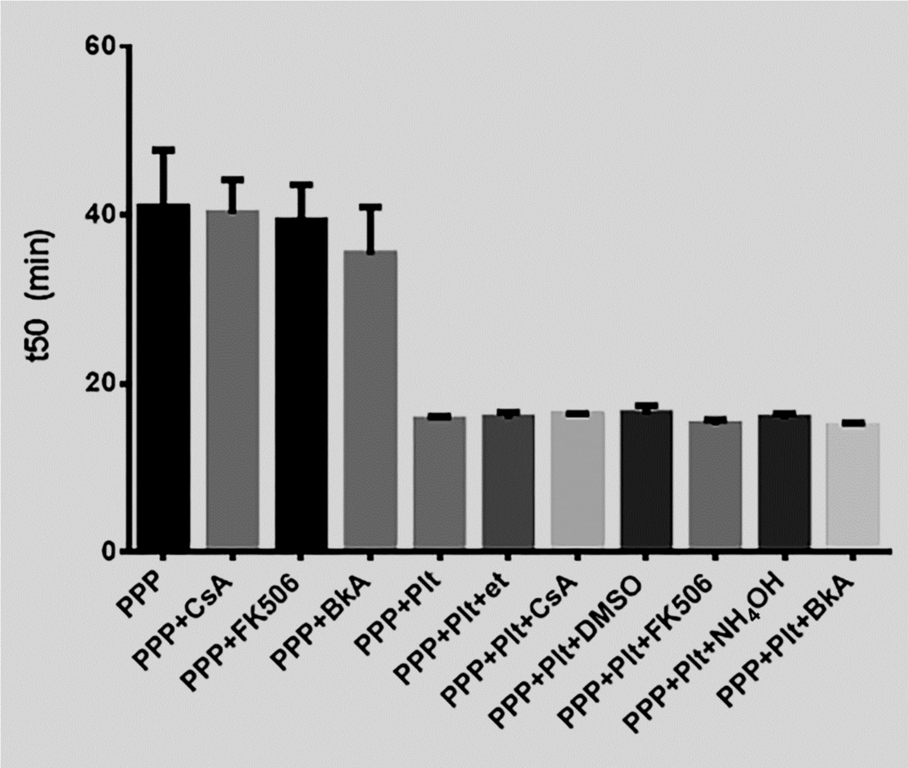

Figure S1. The impact of platelets and modulators of their function on tissue factor induced clotting of plasma. Tissue factor induced clotting was followed by measuring the absorbance at 340 nm. Time to reach half of the maximal absorbance value (t50) was calculated. The figure shows mean t50 values with standard deviation of platelet-poor pooled plasma in the absence and presence of platelets and the indicated modulators or their respective vehicles. Each column represents mean values of eight parallel measurements. Addition of different modulators did not change the t50 values in the presence of platelets

(p>0.05 , Kolmogorov-Smirnov statistical test). PPP: platelet-poor pooled plasma; CsA: Cyclosporin A; BkA: Bongkrekic acid; Plt: platelet; et: ethanol; DMSO: dimethyl-sulfoxide.
